# Patterns of RT-PCR Test Conversion and Implications on Time of Discharge in a District Hospital and a COVID-19 Care Centre in Pali, Rajasthan, India

**DOI:** 10.7759/cureus.27325

**Published:** 2022-07-27

**Authors:** Hazarimal Choudhary, Latika N Sinha, Rashmi Belodu, Sohel Solanki, Hareesh RS Kumar, Ramakrishna Bishnoi

**Affiliations:** 1 Department of General Medicine, Government Medical College and Bangur Hospital, Pali, IND; 2 Department of Preventive and Social Medicine, Government Medical College and Bangur Hospital, Pali, IND; 3 Department of Microbiology, Dr. N. Y. (Nandkumar Yadavrao) Tasgaonkar Institute of Medical Science, Raigad, IND; 4 Department of Anatomy, Government Medical College Barmer and Associated Hospital, Barmer, IND; 5 Department of Forensic Medicine and Toxicology, Government Medical College Barmer and Associated Hospital, Barmer, IND; 6 Department of Pediatrics, Government Medical College and Bangur Hospital, Pali, IND

**Keywords:** covid-19, rt-pcr, district hospital, coronavirus disease, conversion, antigenic

## Abstract

Introduction

Although coronavirus disease 2019 (COVID-19) first appeared in 2019, the symptoms are similar to common viral illnesses, and if undiagnosed or there is a delay in treatment, it may prove fatal because of the virus's propensity to attack the respiratory and cardiovascular system. The antigenic conversion status of reverse transcription-polymerase chain reaction (RT-PCR) was an important criterion for discharge among the COVID-19 patients of the two hospitals in the study.

Aim

The aim of the study was to assess the time taken to antigenic conversion from positive to negative in RT-PCR test for COVID-19 done on patients admitted to the two hospitals where the study took place.

Materials and methods

A prospective cross-sectional study with repeated sampling for antigenic conversion by RT-PCR was done on 117 patients of different age groups admitted to Bangur Hospital and Agarsen Bhavan in Pali, Rajasthan, India, from April 27 to June 30, 2020. Pharyngeal and nasal swabs were analyzed by real-time RT-PCR for COVID-19 infection. The patients' first positive sample was taken as “zero sample”. If the repeat sample taken on the fifth day was positive, sampling was repeated after 48 hours on consecutive days 7, 9, 11, 13, and 15 of admission till the RT-PCR test returned negative.

Results

Among the 117 patients, 92 (78.63%) were treated as mild, 10 (8.54%) were moderately severe, and 15 (12.82%) were very severe requiring ICU care. The median rate of conversion of RT-PCR test (positive to negative) from the day of admission was quite variable as five patients converted to negative by RT-PCR test on day seven of admission, one on day eight of admission, 26 on day nine, 30 on day 10, five on day 11, 13 on day 12, 10 on day 13, 11 on day 14, five on day 15, six on day 16, three on day 17, and one on day 18 of admission.

Conclusion

The study proved that follow-up of patients, prompt and comprehensive treatment, and repeated sampling ensures fast recovery with implications on time to discharge of such patients in a pandemic. The study justified and heralds the message that the inherent immunity of an individual corresponds to the time taken to conversion from positive to negative in the RT-PCR test.

## Introduction

Coronavirus disease 2019 (COVID-19) first appeared in 2019 and is the disease caused by a novel virus called severe acute respiratory syndrome coronavirus 2 (SARS-CoV-2). The World Health Organization (WHO) first learned of this new virus on December 31, 2019, following a report of a cluster of cases of “viral pneumonia” in Wuhan, People’s Republic of China [1]. The common symptoms of COVID-19 are fever, dry cough, and fatigue; associated symptoms are loss of sense of smell and taste, nasal congestion, conjunctivitis, sore throat, headache, muscle or joint pain, different types of skin rash, nausea or vomiting, diarrhea, chills, or dizziness [2]. The severe symptoms include shortness of breath, loss of appetite, confusion, persistent pain, or pressure in the chest, high fever, occasional irritability, confusion, reduced consciousness, anxiety, depression, and sleep disorders [3].

Although COVID-19 symptoms are like those of a common viral illness, if undiagnosed or delayed in treatment it may prove fatal because of the virus's propensity to attack the respiratory and cardiovascular system. Most of the patients (80.9%) who developed symptoms of COVID-19 recovered from the disease without needing hospital treatment; about 13.8% of admissions were severely ill and required oxygen and 4.7% of these were critically ill and needed intensive care with admissions to the ICU [4]. With increasing severity involving the cardiovascular and respiratory systems, oxygen, broad-spectrum antibiotics, and anti-coagulation therapies were the mainstay treatments [5].

Conversion (antigenic conversion from positive to negative) status of reverse transcription-polymerase chain reaction (RT-PCR) was an important criterion for discharge in the COVID-19 patients included in the study. The aim of this study was to evaluate the association between patients' severity of the condition at the time of admission, time taken to antigenic conversion from positive to negative in RT-PCR test for COVID-19 done on patients admitted, and implications on time of discharge from hospital.

## Materials and methods

A descriptive cross-sectional study with repeated sampling for antigenic conversion was done. The convenience sampling was done in 117 COVID-19-positive patients of different age groups, admitted during the period from April 27 to June 30, 2020, in the district administration dedicated COVID-19 Care Center at Agarsen Bhavan and the dedicated COVID-19 hospital at the Government Medical College and Bangur Hospital, Pali, Rajasthan, India. The study was done based on convenience sampling as per available cases during the time.

Study setting

The study population was inpatients of Bangur Hospital, Pali, Rajasthan, India, and Agarsen Bhavan, Pali, Rajasthan, India, admitted from April 27 to June 30, 2020.

Human subject protection considerations

The study proposal was submitted to the Institutional Ethics Committee of Government Medical College and Bangur Hospital, Pali, Rajasthan, and an expedited clearance was obtained considering the emergent need for the study vide letter no. GMC/IEC/2020/102-C3, dated March 3, 2021. Informed consent was taken from every participant in the local vernacular language.

Inclusion criteria

Clinical diagnosis of COVID-19 was made on the basis of relevant epidemiologic history, clinical manifestations, positive findings on High-resolution computed tomography (HRCT) findings wherever available. The patients who had severe breathlessness or/and exhibited febrile symptoms with saturation of peripheral oxygen (SPO2) levels below 94% saturation with or without chest x-ray posteroanterior view having patchy appearance.

Exclusion criteria

The patients suffering from asthma and chronic obstructive pulmonary disease (COPD), and having negative D-dimer and negative x-ray reports were excluded from study.

Method of analysis

As per the standard guidelines issued by the Ministry of Health and Family Welfare and the Indian Council of Medical Research (ICMR) dated March 17, 2020 [6] and following the testing directives issued by the WHO, upper respiratory specimen (pharyngeal and/or nose) swab were obtained and analyzed by real-time RT-PCR for COVID-19 infection. Periods of viral shedding were analyzed by a positive to negative conversion via RT-PCR test. Patient’s first positive sample was taken as “zero sample” and his/her first repeat sample was taken on fifth day of admission; second test was done after 24 hours even for those who tested negative. Two consecutive repeat negative samples were considered as antigenically converted by RT-PCR. For those patients whose repeat sample came positive on day five, subsequent repeat samples were taken after 48 hours on consecutive days 7, 9, 11, 13, and 15 of admission till the RT-PCR test returned negative.

The intervals for repeat testing, antigenic conversion were calculated from the date of first zero sample testing positive. The duration of viral antigenic positivity for a particular patient as considered to be the time from date of first zero sample testing positive to the last positive result for the same patient on a SARS-CoV-2 PCR test before clinical recovery and discharge. After laboratory investigations, supportive therapy was given according to the clinical requirement. In the investigation panel, D-dimer, included C-reactive protein (CRP) test, arterial blood gas (ABG), spike (S) protein, ferritin, and Interleukin-6 (IL-6) was carried out as per the patient’s condition. Liver function test (LFT), complete blood count (CBC), and sugar levels were monitored daily to ascertain to whom Remdesivir was to be administered. The patients were maintained on oxygen therapy in severe breathlessness. Treatment was given to patients whose SpO2 was below 94% and with a respiratory rate of more than 24/minutes (Table 1).

**Table 1:** Supportive therapy or treatment given to COVID-19 patients. Tab: tablet; IV: intravenous; mg: milligram; ml: milliliter; OD: once a day; BD: twice a day; SOS: si opus sit/if needed; LFT: liver function test; RFT: renal function test; CBC: complete blood count; COVID-19: coronavirus disease 2019

Sr. no.	Medicine	Route	Quantity	Frequency
1.	Injection- Piperacillin + Tazobactam	IV	4.5 mg	BD for 5 days
2.	Injection- Ranitidine	IV	25 mg (2 ml)	BD for 5 days
3.	Injection- Metoclopramide	IV	10 mg (2 ml)	BD/ (SOS)
4.	Tablet - Vitamin C	Oral	500 mg.	2 Tab. BD
5.	Tablet - Zinc	Oral	20 mg.	BD
6.	Tablet- Aspirin	Oral	150 mg	OD
7.	Tablet- Ivermectin	Oral	12 mg.	OD X 3 days
8.	Tablet- Paracetamol	Oral	500 mg.	1 SOS
9.	Tablet- Levocetirizine Montelukast	Oral	5 mg + 10 mg	OD
10.	Injection 100 CC N.S + Remdesivir	IV	200 mg	For first day
11.	Injection 100 CC NS + Remdesivir	IV	100 mg	for next 4 days
12.	Injection Low Molecular weight heparin (LMWH)	IV	60 mg	OD for 5 days after LFT, RFT, CBC are normal.

Patient outcome

Patients' condition was noted as mild, moderate, and severe along with the number of days taken to recover. Patients were categorized as follows [7]: (i) Mild symptomatic patients meeting the case definition for COVID-19 without evidence of viral pneumonia or hypoxia, including SpO2 > 90%; (ii) Moderate symptomatic patients with clinical signs of pneumonia (fever, cough, dyspnoea, fast breathing) but no signs of severe pneumonia, including SpO2 ≥ 90%; (iii) Severely symptomatic patients with clinical signs of pneumonia (fever, cough, dyspnoea, fast breathing) plus severe respiratory distress or SpO2 < 90% on room environment. 

Statistical analysis

The statistical analysis in regards to the proportion of cases in each category of the severity of the clinical condition at the time of admission and trends in antigenic conversion from the date of admission was done by using IBM SPSS Statistics for Windows, Version 19.0 (Released 2010; IBM Corp., Armonk, New York, United States). Descriptive statistical analysis was used for summarizing the data using mean, median, and frequency.

## Results

**Figure 1:**
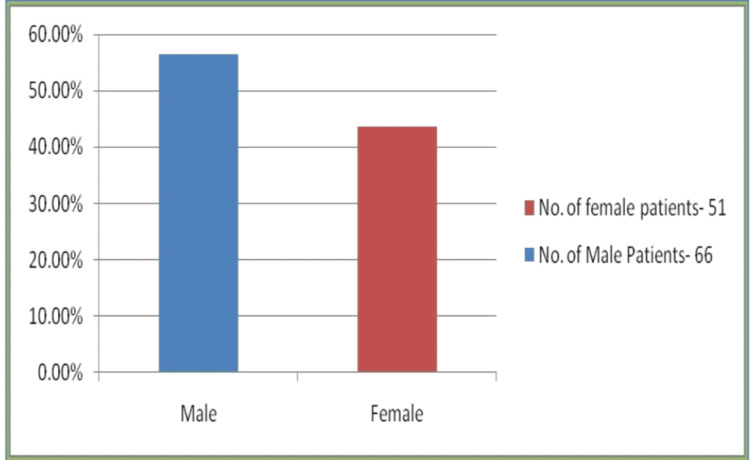
Gender-wise distribution of COVID-19 patients (n=117) COVID-19: coronavirus disease 2019

The conditions of illness of the patients under study were categorized as mild in 92 (78.63%) requiring only oxygen support, 10 (8.54%) were in the moderately severe category requiring oxygen support with antibiotics, and 15 (12.82%) were severely ill requiring ICU care. Among the 117 patients, 66 (56.41%) were males and 51 (43.58%) were females, the median age being 28 years (Figure 1).

**Figure 2:**
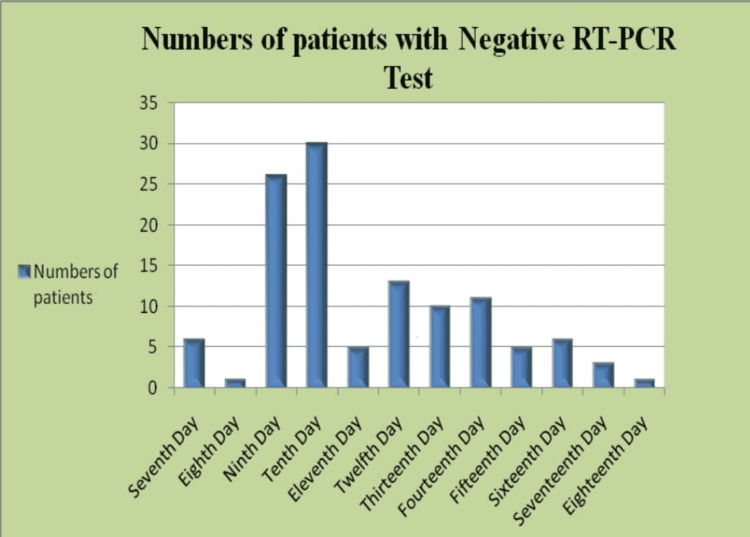
Antigenic conversion of COVID-19 patients by RT-PCR from the day of admission Days of conversion (Number of patients converted): Day 7 (6), Day 8 (1), Day 9 (26), Day 10 (30), Day 11 (5), Day 12 (13), Day 13 (10), Day 14 (11), Day 15 (5), Day 16 (6), Day 17 (3), and Day 18 (1).

Patients were admitted to the hospital on the same day the zero samples returned with positive test results. On subsequent sampling, no patient was found converted negative till day six of admission, i.e. within five days from the day of admission. The positive to negative RT-PCR test conversion of patients from the day of admission were as follows: six (5.12%) on day seven of admission, one (0.85%) on day eight of admission, 26 (22.22%) on day nine, 30 (25.64%) on day 10, being highest recovery, five (4.27%) on day 11, 13 (11.11%) on day 12, 10 (8.54%) on day 13, 11 (9.4%) on day 14, five (4.27%) on day 15, six (5.12%) on day 16, three (2.56%) on day 17, and one (0.85%) on day 18 of admission. The numbers of patients with the dates of admission and antigenic conversion of RT-PCR test is shown in Figure 2 and Table 2.

**Table 2:** Number of patients with date of admission and conversion in the hospital. DOA: date of admission; NOP: number of patients; DOA: date of admission; DOC: date of antigenic conversion; N/A: not applicable

S. N.	Days of Conversion (DOA- DOC)	Number of patients with date of admission and recovery						Total number of antigenically converted patients
1.	Day 7 of admission	NOP	06	N/A	N/A	N/A	N/A	06
		DOA	13/05/20	N/A	N/A	N/A	N/A	
		DOC	19/05/20	N/A	N/A	N/A	N/A	
2.	Day 8 of admission	NOP	01	N/A	N/A	N/A	N/A	01
		DOA	17/05/20	N/A	N/A	N/A	N/A	
		DOC	24/05/20	N/A	N/A	N/A	N/A	
3.	Day 9 of admission	NOP	01	02	06	07	10	26
		DOA	03/05/20	08/05/20	05/05/20	07/05/20	17/05/20	
		DOC	11/05/20	16/05/20	13/05/20	15/05/20	25/05/20	
4.	Day 10 of admission	NOP	3	3	2	5	17	30
		DOA	06/05/20	10/05/20	11/05/20	13/05/20	17/05/20	
		DOC	15/05/20	19/05/20	20/05/20	22/05/20	26/05/20	
5.	Day 11 of admission	NOP	1	1	1	2	N/A	05
		DOA	29/04/20	06/05	09/05	16/05	N/A	
		DOC	09/05/20	16/05	19/05	26/05	N/A	
6.	Day 12 of admission	NOP	6	2	1	4	N/A	13
		DOA	28/04/20	10/05/20	14/05/20	15/05/20	N/A	
		DOC	09/05/20	21/05/20	25/05/20	26/05/20	N/A	
7.	Day 13 of admission	NOP	06	02	01	01	N/A	10
		DOA	27/04/20	07/05/20	10/05/20	13/05/20	N/A	
		DOC	09/05/20	19/05/20	22/05/20	25/05/20	N/A	
8.	Day 14 of admission	NOP	01	02	02	06	N/A	11
		DOA	27/04/20	28/04/20	06/05/20	13/05/20	N/A	
		DOC	10/05/20	11/05/20	19/05/20	26/05/20	N/A	
9.	Day 15 of admission	NOP	01	02	02	N/A	N/A	05
		DOA	25/04/20	27/04/20	06/05/20	N/A	N/A	
		DOC	09/05/20	11/05/20	20/05/20	N/A	N/A	
10.	Day 16 of admission	NOP	01	02	03	N/A	N/A	06
		DOA	28/04/20	06/05/20	07/05/20	N/A	N/A	
		DOC	13/05/20	21/05/20	22/05/20	N/A	N/A	
11.	Day 17 of admission	NOP	01	01	01	N/A	N/A	03
		DOA	27/04/20	29/04/20	06/05/20	N/A	N/A	
		DOC	13/05/20	15/05/20	22/05/20	N/A	N/A	
12.	Day 18 of admission	NOP	01	N/A	N/A	N/A	N/A	01
		DOA	03/05/20	N/A	N/A	N/A	N/A	
		DOC	20/05/20	N/A	N/A	N/A	N/A	
Total Number of antigenically converted Patients								117

A hundred patients (85%) had self-limiting illness. The median period of conversion was nine days and 91% patients’ RT-PCR tests were negative by days 6-15 after prescribed treatment. It was seen that patients with age <60 years had earlier antigenic conversion rates than patients >60 years of age and a 4-7% variation was estimated between the two age strata (Table 3).

**Table 3:** Association of age with days to antigenic conversion. n: total number of patients converted; n1: numbers of patients <60 years (21 to 60 years of age); n2: numbers of patients >60 years of age; NIL: patient not converted to corresponding day of admission

Days of Conversion	Total (n=117)	Age distribution (in years)				
		<60 years (n_1_=94)				>60 years
		21-30	31-40	41-50	51-60	(n_2_=23)
Day 7 of Admission	06	2 (33.33%)	1 (16.66%)	1 (16.66%)	1 (16.66%)	1 (16.66%)
Day 8 of Admission	01	1 (100%)	NIL	NIL	NIL	NIL
Day 9 of Admission	26	11 (42.3%)	4 (15.38%)	4 (15.38%)	3 (11.53%)	4 (15.38%)
Day 10 of Admission	30	8 (26.66%)	6 (20%)	5 (16.66%)	5 (16.66%)	6 (20%)
Day 11 of Admission	05	3 (60%)	1 (20%)	NIL	1 (20%)	NIL
Day 12 of Admission	13	7 (53.84%)	1 (7.69%)	NIL	3 (23%)	2 (15.38%)
Day 13 of Admission	10	2 (20%)	4 (40%)	1 (10%)	NIL	3 (30%)
Day 14 of Admission	11	2 (18.18%)	4 (36.36%)	1 (9%)	1 (9%)	3 (27.27%)
Day 15 of Admission	05	NIL	1 (20%)	NIL	3 (60%)	1 (20%)
Day 16 of Admission	06	NIL	NIL	2 (33.33%)	3 (50%)	1 (16.66%)
Day 17 of Admission	03	NIL	NIL	1 (33.33%)	1 (33.33%)	1 (33.33%)
Day 18 of Admission	01	NIL	NIL	NIL	NIL	1 (100%)

## Discussion

Coronavirus disease 2019 was first reported in Wuhan, China, in December 2019 [1,2]. By August 2020, COVID-19 had affected over 18 million persons, spread among 216 countries and regions, and caused nearly 700,000 deaths worldwide [8]. Knowledge of viral PCR positivity patterns, duration, and neutralizing antibody responses is essential for developing an epidemiologic manipulation strategy, selecting the precise antiviral treatment, and making a knowledgeable choice on vaccinations. Many centers have tried to understand the SARS-CoV-2 viral kinetics and positivity [9,10].

Wolfel et al. observed that seroconversion by day seven was seen in only half the patients but after 14 days all the patients showed seroconversion but this was not necessarily followed by a rapid decrease in viral load; also stated that RT-PCR of both oral and nasopharyngeal swab specimens showed no discernible differences in viral loads or detection rates [11]. In the study, the virus was readily isolated during the first week of symptoms from a considerable fraction of samples (16.6% of swabs and 83.3% of sputum samples), no isolates were obtained from samples taken after day eight in spite of ongoing high viral loads. They studied virus isolation from stool samples taken between day six and day 12 and found that it was not very successful, irrespective of viral RNA concentration. It is known that virus isolation depended on viral load and samples that contained less than 106 copies per reaction per sample never yielded an isolate.

Further, it was seen that seroconversion in half of the patients occurred by day nine and in all patients by day 14. Viruses isolated after day seven were minimum in the study by Corman et al. Neutralizing antibodies detected in the titers did not suggest any correlation with clinical courses [12]. The trends seen in the studies mentioned solicited the need to further systematically analyze the correlations between viral dynamic PCR positivity, seroconversion, and disease severity in our center.

The conversion trends seen in our study were more or less similar to the findings in the studies by Liu et al. and Li et al. [13,14], in which predictors for conversion have been studied and the median period of conversion from the day of admission was nine days, which is around two days more than the study done in. Liu et al. also found that SARS-CoV-2 persisted and was detectable till day 63 after symptom onset [15]. In India, a study by Yadav et al. showed seroconversion up to 80% in 21 days [16]. These studies have highlighted that long after the clinical symptoms resolved and seroconversion developed, prolonged virus shedding continues and proposed the possibility of a prolonged contagious period and recommended continued and further investigation to better control the epidemics.

Li et al. also observed that the basic reproductive number was 2.2 in infections with COVID-19 in the earliest phase of the outbreak with a mean interval of 7.5 days. There was an average delay of five to six days between infection and illness onset, with an upper limit of around 11-14 days. They also noticed that from onset of illness to laboratory confirmation added 10 days on average. While patients get discharged after 10 days subsequent to symptom arrival or a febrile span of three days (whichever is later) without a negative RT-PCR report in India, China followed a discharge guideline of two consecutive negative tests within 24 hours and European Union had a discharge strategy of three days asymptomatic span or appearance of IgG antibody in respiratory samples [17,18].

 Upon inhalation, the viral particles use “S” protein to latch into host cells and slowly infiltrate the upper portion of the respiratory tract. The primary role is to bind to the angiotensin-converting enzyme 2 (ACE-2) receptor, which is present in the epithelial cells, especially vascular epithelial cells and macrophages in the lungs. This results in the compromised pulmonary ACE-2 function of the lung and that leads to lung injury over time. This event affects blood pressure and fluid and electrolyte balance. Injury to lung tissue leads to respiratory distress, which impairs the cardiovascular system. In severe cases, multiple organ failure followed by the mortality of patients was also reported.

When our body encounters foreign micro-organisms, it activates the immune system, which provides a specific defense against any invading pathogens. Moreover, immunity is acquired by a nutritious diet and healthy environmental factors. The patients who recovered earlier had high immunity and administration of broad-spectrum antibiotics along with vitamin supplementation [19]. Apart from antiviral and antibiotics, there is a dire need for vaccination in each and every person because vaccine triggers the immune system and induces an effective immune response. The purpose of vaccination is to induce protection against an infectious agent without causing significant disease.

Limitations of the study

The sample size was less due to the short duration of the wave, which lasted for two to three months in the area under study and cases declined suddenly. Serological testing for IgG and IgM was not performed due to constraints of time, sample load in the lab, and manpower in the pandemic situation.

## Conclusions

The trends in antigenic conversion reflected in this study lead to the conclusion that the time taken to convert from positive to negative in the RT-PCR test had a median of seven days and a range of 6-17 days with no case remaining positive after 17 days. The median age in the patients was 28 years and gender was predominantly male reflecting the activity of this age group and the risk of exposure in a pandemic situation. It was also observed that the time to testing negative by PCR depends on the inherent immunity of the individual and is variable. Mild cases could be missed and lead to further spread in the community emphasising the need for intensive as well as voluntary testing in a pandemic situation and a watch over progress of symptoms and testing negative. Awareness campaigns on voluntary testing and symptoms of COVID-19 in the public domain play a large role in this aspect. There was no mortality in the group under study and this also points to a favourable immune response in the group.

This study generates a lesson that more awareness among the people is needed to follow the norms of COVID-19 appropriate behavior including social distancing, wearing masks, and maintaining personal hygiene. Above all, vaccination must be mandatory because it ensures cellular and humoral immunity against fatal diseases.
